# Safety, efficacy, and immunogenicity of an inactivated influenza vaccine in healthy adults: a randomized, placebo-controlled trial over two influenza seasons

**DOI:** 10.1186/1471-2334-10-71

**Published:** 2010-03-17

**Authors:** Lisa A Jackson, Manjusha J Gaglani, Harry L Keyserling, John Balser, Nancy Bouveret, Louis Fries, John J Treanor

**Affiliations:** 1 Group Health Research Institute, Seattle, Washington, USA; 2 Section of Pediatric Infectious Diseases, Scott & White Memorial Hospital and Clinic, Texas A&M University Health Science Center College of Medicine, Temple, Texas, USA; 3 Division of Pediatric Infectious Diseases, Emory University School of Medicine, Atlanta, Georgia, USA; 4 Veristat, Inc, Holliston, Massachusetts, USA; 5 GlaxoSmithKline Biologicals, Laval, Quebec, Canada; 6 Variation Biotechnologies Inc, Gatineau, Québec, Canada; 7 GlaxoSmithKline Biologicals, Columbia, Maryland, USA; 8 Infectious Diseases Unit, University of Rochester Medical Center, Rochester, NY, USA

## Abstract

**Background:**

Seasonal influenza imposes a substantial personal morbidity and societal cost burden. Vaccination is the major strategy for influenza prevention; however, because antigenically drifted influenza A and B viruses circulate annually, influenza vaccines must be updated to provide protection against the predicted prevalent strains for the next influenza season. The aim of this study was to assess the efficacy, safety, reactogenicity, and immunogenicity of a trivalent inactivated split virion influenza vaccine (TIV) in healthy adults over two influenza seasons in the US.

**Methods:**

The primary endpoint of this double-blind, randomized study was the average efficacy of TIV versus placebo for the prevention of vaccine-matched, culture-confirmed influenza (VMCCI) across the 2005-2006 and 2006-2007 influenza seasons. Secondary endpoints included the prevention of laboratory-confirmed (defined by culture and/or serology) influenza, as well as safety, reactogenicity, immunogenicity, and consistency between three consecutive vaccine lots. Participants were assessed actively during both influenza seasons, and nasopharyngeal swabs were collected for viral culture from individuals with influenza-like illness. Blood specimens were obtained for serology one month after vaccination and at the end of each influenza season's surveillance period.

**Results:**

Although the point estimate for efficacy in the prevention of all laboratory-confirmed influenza was 63.2% (97.5% confidence interval [CI] lower bound of 48.2%), the point estimate for the primary endpoint, efficacy of TIV against VMCCI across both influenza seasons, was 46.3% with a 97.5% CI lower bound of 9.8%. This did not satisfy the pre-specified success criterion of a one-sided 97.5% CI lower bound of >35% for vaccine efficacy. The VMCCI attack rates were very low overall at 0.6% and 1.2% in the TIV and placebo groups, respectively. Apart from a mismatch for influenza B virus lineage in 2005-2006, there was a good match between TIV and the circulating strains. TIV was highly immunogenic, and immune responses were consistent between three different TIV lots. The most common reactogenicity events and spontaneous adverse events were associated with the injection site, and were mild in severity.

**Conclusions:**

Despite a good immune response, and an average efficacy over two influenza seasons against laboratory-confirmed influenza of 63.2%, the pre-specified target (lower one-sided 97.5% confidence bound for efficacy > 35%) for the primary efficacy endpoint, the prevention of VMCCI, was not met. However, the results should be interpreted with caution in view of the very low attack rates we observed at the study sites in the 2005-2006 and 2006-2007, which corresponded to relatively mild influenza seasons in the US. Overall, the results showed that TIV has an acceptable safety profile and offered clinical benefit that exceeded risk.

**Trial registration:**

NCT00216242

## Background

Annual epidemics of influenza due to influenza A and B viruses remain a substantial cause of morbidity and mortality worldwide, particularly among vulnerable groups such as people aged ≥ 65 years, children aged <2 years, and people with chronic medical conditions [1-3]. In addition to these identified risk groups, influenza is responsible for a substantial burden of illness, absenteeism, and resultant societal costs among otherwise healthy working adults [4-7]. Trivalent inactivated influenza vaccines (TIV) containing antigens of two influenza A strains (one A/H1N1 and one A/H3N2), in combination with antigens of one influenza B strain, provide the current standard for influenza prevention. Because one or more new antigenically drifted variants circulate annually, the vaccines must be updated to provide optimal protection against the predicted prevalent strains for the next influenza season [8-10]. The World Health Organization (WHO), as well as the US Food and Drug Administration (FDA) Center for Biologics Evaluation and Research, provide annual guidance for strain selection based on new drift variants detected through a global influenza surveillance network [8,9].

Clinical trial data have repeatedly shown that TIVs can be protective against seasonal influenza, including seasons when the surface antigens of the prevalent virus(es) match the vaccine strains, and some seasons when a drifted strain circulates, although efficacy can be reduced as a result of substantial antigenic drift [8,11-14]. However, many TIV studies have been either too small to provide narrow confidence limits about the point estimates for efficacy, or have used serological criteria to define the influenza infection endpoint, which unlike virus detection by culture or molecular methods, may bias the results in favor of the vaccine because detection of infection by seroconversion may be adversely affected by prior elevation of baseline titers in vaccinated, but not unvaccinated subjects [15,16]. Furthermore, marked variations in efficacy estimates have been observed from season to season, even when the same investigators have applied identical methods to evaluate the same type of vaccine formulation in consecutive years [12,13].

In this paper, we describe an efficacy, safety, and immunogenicity study of a trivalent inactivated split virus influenza vaccine (TIV; marketed, depending on the countries, as *Fluviral*^®^, *FluLaval*™, or *Griplaval*™ trademarks of the GlaxoSmithKline group of companies). The product, first licensed in Canada in 1992, has undergone several process refinements, including introduction of a concentration step, homogenization, and sterile filtration. Following clinical studies of safety and immunogenicity in adults conducted in Canada and the US in the 2004-2005 influenza season, the product received accelerated approval from the FDA in 2006 [17]. The randomized, placebo-controlled study reported in this paper is part of the post-approval clinical trial program, conducted to confirm manufacturing consistency, and to further assess the clinical benefits of this TIV formulation using culture-confirmed efficacy endpoints[17].

The study included healthy adults aged 18 to 49 years in the US, and in order that a placebo control could be ethically employed, high-risk patients for whom annual influenza vaccine is recommended by the Advisory Committee on Immunization Practices (ACIP) were not eligible for inclusion [18]. The primary clinical endpoint was based upon rates of culture-confirmed influenza illness caused by A and/or B virus strains antigenically matching those in the vaccine. The study was conducted over the 2005-2006 and 2006-2007 influenza seasons.

## Methods

### Design and participants

This was a randomized, double-blind, placebo-controlled study of the efficacy of TIV in prevention of vaccine-matched, culture-confirmed influenza (VMCCI) conducted in the 2005-2006 and 2006-2007 influenza seasons in the US.

The original primary outcome measure defined by the study protocol was the average vaccine efficacy over two consecutive seasons in the prevention of culture-confirmed influenza. In correspondence following the 2005-2006 season, the FDA Center for Biologics Evaluation and Research noted that the season was marked by a significant frequency of circulation of influenza virus strains that were antigenically-drifted from those in the vaccine, and required that the protocol be modified to assess the average efficacy against VMCCI across both seasons as the primary measure of vaccine efficacy.

Male and female volunteers aged 18 to 49 years inclusive were eligible to participate if they were clinically healthy, understood the study procedures, had access to telephone contact throughout study, and provided informed written consent. In Season 1, eligible participants were enrolled at 37 centers, and in Season 2, eligible participants were enrolled at 44 centers.

Exclusion criteria included: a significant acute or chronic, or medical or psychiatric illness requiring institution of new medical or surgical treatment, or a significant dose alteration for uncontrolled symptoms or drug toxicity within 3 months; diagnosis of cancer, or treatment for cancer, within 3 years; systolic blood pressure ≥ 140 mmHg, diastolic blood pressure ≥ 90 mmHg; a health condition placing the potential subject in a risk group recommended for routine influenza immunization by Advisory Committee on Immunization Practices (ACIP), e.g. chronic pulmonary (including asthma), cardiovascular, renal, hepatic, hematological, or metabolic disorders (including diabetes mellitus); immunosuppressive illness, recent/ongoing receipt of immunosuppressive therapy, or human immunodeficiency virus infection [18]; recent administration of other vaccine or immunoglobulin, contraindication to influenza vaccine, or previous influenza vaccination within 9 months. A negative pregnancy urine test at screening was required for women of childbearing potential before inclusion into the study, and women were required to use reliable contraception throughout the study. Individuals employed in professions prone to influenza transmission to or from high-risk populations, and individuals living in the same household as an immunocompromised person were not eligible to participate (also based on ACIP recommendations [18]).

In order to allow an independent assessment of efficacy in each season, participants who were enrolled in the study during 2005-2006 (Season 1) were not eligible for enrollment during 2006-2007 (Season 2).

The study was conducted in accordance with Good Clinical Practice, US regulatory requirements, and the Declaration of Helsinki. This protocol and consent for this multi-centered study were approved by six (6) independent ethics committees/institutional review boards as required to provide ethical oversight for 44 sites. The names of these bodies are on file with the corresponding author.

### Vaccine allocation and administration

Treatment allocation was determined by blocked, stratified randomization with a 1:1 distribution to TIV or placebo; randomization was stratified by study center, age (18-34 and 35-49 years), and the subject's report of previous recent receipt (within ≤ 2 years) of TIV. Each study center had a pre-determined sequence of randomization numbers which were allocated sequentially to eligible subjects. Participants were allocated equally among 3 different vaccine lots. Clinic staff (excluding the nurse giving the vaccine), were blinded to the treatment group until the study was complete.

Participants received a single injection of TIV (*FluLaval*™, a trademark of the GlaxoSmithKline group of companies; manufactured by ID Biomedical Corporation of Quebec [IBD-Q], Canada), or saline placebo injection. Each 0.5 mL dose of TIV contained 15 μg of hemagglutinin (HA) antigen of each recommended influenza strain. Antigens for Season 1 (2005-2006) were A/New Caledonia/20/1999 (H1N1), A/New York/55/2004 (H3N2, A/California/7/2004-like), and B/Jiangsu/10/2003 (B/Shanghai/361/2002-like). Antigens for Season 2 (2006-2007) were A/New Caledonia/20/1999 (H1N1) virus, A/Wisconsin/67/2005 (H3N2), and B/Malaysia/2506/2004. Three consecutive vaccine lots were used in each season.

### Outcomes

The primary endpoint was the average efficacy over Seasons 1 and 2 for the prevention of culture-confirmed influenza illness due to influenza A or B virus strains antigenically matching those in the vaccine (i.e. vaccine-matched, culture-confirmed influenza; VMCCI). The case definition of VMCCI required the presence of influenza-like illness (ILI), defined as symptoms that interfered with normal daily activities and that included cough, and at least 1 additional symptom from among fever (oral temperature >37.7°C/99.9°F), headache, myalgia and/or arthralgia, chills, rhinorrhea/nasal congestion, and sore throat. Participants meeting the definition for ILI and with concurrent isolation from a nasopharyngeal swab of an influenza A and/or B virus isolate antigenically matching a vaccine strain for the relevant year were considered to be cases of VMCCI.

Co-primary safety endpoints were the assessment of solicited local and general reactogenicity events occurring between 0 and 3 days of vaccination, and unsolicited spontaneous adverse events (AEs) for a minimum of 135 days post-vaccination.

The average efficacy over Seasons 1 and 2 against culture-confirmed influenza illness (CCI), or laboratory-confirmed influenza illness (LCI) were secondary endpoints. CCI was defined as ILI with any influenza A or B virus isolate by culture, and LCI was defined as one or both of CCI or ILI with a 4-fold increase in hemagglutination-inhibiting (HI) serum antibody titers to a circulating influenza virus strain between Day 21 (± 4 days) post-vaccination and Final Visit specimens obtained after the end of the influenza season. The average efficacy across both seasons was also assessed according to age, prior vaccination status, and gender; but no attempt was made to provide statistical power for hypothesis tests within these subgroups.

Immunogenicity endpoints were HI assay-based Day 21 seroconversion and seroprotection rates, geometric mean fold-rises (GMFR) between Day 0 and Day 21, and lot equivalence according to reciprocal GMTs at Day 21 in Season 1.

### Surveillance

The surveillance period in each season was ~15 weeks. Surveillance for influenza was conducted between 14 November 2005 through 30 April 2006 (Season 1), and 13 November 2006 through 30 April 2007 (Season 2). Throughout the influenza season, participants were instructed to report symptoms meeting the definition of ILI via a study-specific, toll-free telephone number within 48 hours of symptom onset, and to record temperatures and symptoms during ILI episodes. This passive surveillance method was accompanied by active surveillance in which ILI symptoms were solicited during weekly outbound telephone contact. Local visiting nurses were dispatched to participants fulfilling the definition of ILI within 24 hours of reporting, and obtained nasopharyngeal and oropharyngeal swabs for viral culture. To ensure that influenza illnesses were detected, participants could be swabbed more than once.

### Antigenic characterization and serology

Blood samples were taken before vaccination on Day 0, and at the next study visit on Day 21 (± 4 days), and at the Final Visit, which was within 4 weeks following the end of the surveillance period. Following serum separation, samples were frozen until analysis. Combined nasopharyngeal and oropharyngeal swab samples from participants reporting ILI were refrigerated and transported to a central laboratory (Covance Central Laboratory Services, Inc., Indianapolis, US) for influenza virus culture and assessment of CCI. Samples were cultured using primary monkey kidney cells, and influenza A and B viruses were identified using immunofluorescent staining with influenza-specific monoclonal antibodies. Influenza A viruses were further sub-typed by immunofluorescence using H1 and H3 specific reagents.

Detailed antigenic characterization and vaccine match analysis (i.e. VMCCI) of the influenza viral isolates was determined by serologic methods in the laboratory of Dr John Treanor (University of Rochester Medical Center [URMC], New York, US), using ferret sera for influenza A virus strains (kindly provided by Dr Alexander Klimov, CDC, Atlanta, Georgia, US), or WHO typing sera for B lineages. Isolates were considered to be 'vaccine matching' if there was a ≤ 4-fold difference in the HI titer of the subject isolate strain and the vaccine prototype strain with reference anti-serum [19]. Testing included TIV virus strains, and drifted influenza A virus strains or alternate-lineage influenza B virus strains circulating in each year. The isolate type and subtype were also confirmed using reverse transcription polymerase chain reaction (RT-PCR; GSK Biologicals, Rixensart, Belgium). RT-PCR confirmed the isolate type and subtype determined by immunofluorescence and/or HI testing for 100% of isolates.

To evaluate the endpoint of LCI, Day 21 and Final Visit sera of all participants who reported ILI, irrespective of culture-confirmation, were tested for seroconversion. Immunogenicity of the vaccine was examined by testing of Day 0 and Day 21 serum samples in a randomly selected subset of participants. Immunogenicity was assessed using established HI assay methods performed at the IBD-Q analytic laboratory (IBD-Q/GlaxoSmithKline Biologicals North America Clinical Immunology Laboratory, Laval, Quebec, Canada) [20].

### Reactogenicity and safety

After the injection, participants were observed for 30 minutes for local and/or systemic reactogenicity, and oral temperature was taken. Participants received memory aids to record the severity of injection site reactions, reactogenicity events, and general solicited adverse events (AEs). Definitions of events and severity grades were provided with the memory aids. Participants reported immediate reactogenicity data (within 30 minutes of dosing) before leaving the clinic, and solicited reactogenicity symptoms (occurring on Day 0 from 30 minutes post-vaccination to the evening of Day 3) no later than 8 days post-vaccination via an Interactive Voice Response System (IVRS).

Data on AEs and serious adverse events (SAEs) were reported throughout the entire study period. AEs were captured using electronic case report forms and coded by preferred term and primary system organ class (SOC) using the Medical Dictionary for Regulatory Activities (MedDRA).

### Sample size

Sample size calculations were based on the Farrington-Manning method, as implemented in the PASS software package. We assumed a vaccine efficacy of 70% and a rate of culture-confirmed influenza (CCI) of 2.0% in placebo recipients in each season. The originally planned sample size was selected to provide 80% power in a one-sided test with type 1 error = 0.025 to reject the null hypothesis that the average vaccine efficacy was less than 35%. An adjustment of 10% was applied to account for potential drop-outs, resulting in a target of 3187 participants per study arm across both seasons (total 6374). After the end of Season 1, at the request of the regulatory authority (US FDA, Center for Biologics Evaluation and Research), the primary study endpoint was changed from CCI to vaccine-matching, culture-confirmed influenza illness (VMCCI).

As specified in the protocol, at the end of Season 1 an unblinded, independent statistician, who was not otherwise involved in the analysis, evaluated the actual rate of CCI detected during Season 1. This had originally been planned to ensure that the estimated sample size across both seasons would confer sufficient power to demonstrate average efficacy against CCI with the requisite degree of precision and to allow adjustment of the sample size in Season 2 if needed. Given the change in the primary endpoint, this determination also involved estimation of the proportion of influenza isolates identified from the placebo group that would be antigenically matched to the vaccine strains, to ensure that the study would be adequately powered for the endpoint of VMCCI. Therefore, the statistician assumed that 66.8% of influenza isolates identified in the placebo group in Season 1 (all of which had not yet been sub-typed) were antigenically matched to the vaccine strains, based on 2005-2006 US surveillance data, and that 80% of influenza isolates identified from the placebo group in Season 2 would be antigenically matched to the vaccine strains, based on the mean and median values of US surveillance results for the preceding 6 years [21]. Based on these estimations and the actual CCI attack rate identified in Season 1, the enrollment target for Season 2 was increased from 3187 to 3900 participants.

### Statistical analysis

The primary analysis was performed on the Per-Protocol (PP) set including participants who received the treatment to which they were randomized, responded to ≥ 1 post-vaccination active surveillance telephone call, and with no major protocol deviations considered to affect the efficacy or immunogenicity data. Exclusions from the PP set were determined and documented before unblinding. The possible introduction of biases by use of the PP set was assessed by repeating efficacy analyses in the ITI set, which was the PP set plus participants with protocol deviations and treatment errors, and analyzed as randomized. Immunogenicity was evaluated in a randomly selected subset of participants (immunogenicity set).

The primary efficacy analysis was based on the average efficacy over Seasons 1 and 2 and assessed the null hypothesis that the average efficacy in the actively immunized group was ≤ 35%, against the alternative hypothesis that average efficacy was >35%. Average efficacy was defined as 1 - v(R_1_R_2_) × 100 where R_1 _and R_2 _were the relative risks of a given disease endpoint in Seasons 1 and 2, respectively. A one-sided 97.5% CI was constructed for the average efficacy, and the TIV efficacy target was to be established if the lower bound of the CI was >35%.

The seroconversion rate was defined as the proportion of participants with a ≥ 4-fold increase in reciprocal HI titer at Day 21 versus Day 0, or a reciprocal HI titer of ≥ 40 from a starting value <10; seroprotection rate was the proportion of participants with HI titers ≥ 1:40 at Day 21. The geometric means of reciprocal HI titers (GMTs) were calculated for Day 0 and Day 21 samples, and the geometric mean fold-rises (GMFR; geometric mean of the within-subject fold increases from Day 0 to 21) were also calculated. Lot equivalence was assessed using reciprocal GMTs at Day 21 in the immunogenicity set in Season 1. The Zmin method of Wiens and Iglewicz (WI) was used to assess overall lot equivalence with a two-sided 0.05 significance level and power of ≥90% [22]. One-sided 97.5% lower confidence bounds (identical to two-sided 95% lower confidence bounds) on the rate of seroconversion, seroprotection, and GMT were calculated with each Season.

The safety set included participants who received any study treatment and had any post-vaccination safety data. If an incorrect treatment was conclusively documented, participants in the safety set were analyzed based on the treatment they actually received. The incidence of immediate complaints, reactogenicity events from Day 0 to Day 3, and unsolicited AEs were compared between the TIV and placebo groups using Fisher's exact tests. Any reactogenicity event at grade 2 or 3 with an incidence of >10% was assessed using an extended Mantel-Haenszel test. Statistical analyses of safety were performed at the two-sided significance level of 0.05, with no correction for multiple comparisons.

## Results

### Participants

**Figure 1:**
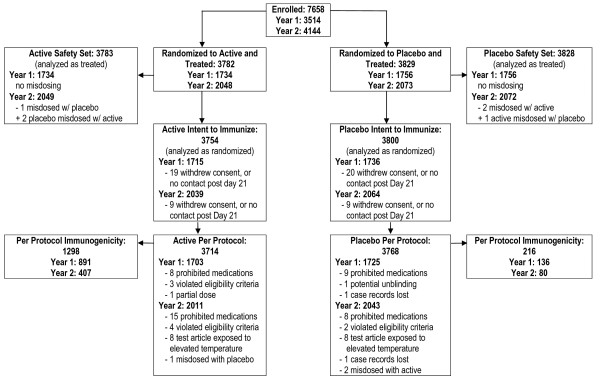
**Enrollment and Inclusion in Analysis Sets over Two Seasons**.

Participant enrollment in Season 1 began 17 September 2005 and follow-up ended 31 May 2006, and enrollment in Season 2 began 16 October 2006 and follow-up ended 6 June 2007. A total of 7611 participants received treatment; 3783 participants received TIV, and 3828 received placebo. A total of 7219 (95%) of vaccinated participants completed the study (Figure 1). The PP set included 3714 participants in the TIV group, and 3768 participants in the placebo group. No participants with culture-confirmed influenza illness were excluded from the primary analysis.

There were no significant differences between the treatment groups for any baseline characteristic (Table 1). In both groups, 57% of participants were aged 18-34 years, and 43% were aged 35-49 years.

**Table 1:** Demographic and clinical characteristics of study participants.

	Vaccine N = 3783	Placebo N = 3828
Mean age, years (SD)	32.7 (9.19)	32.7 (9.14)
1. Male, n (%)	1465 (39)	1520 (40)
Age stratum, n (%)		
Age 18 - 34	2153 (57)	2181 (57)
Age 35 - 49	1630 (43)	1647 (43)
Race, n (%)		
Caucasian	3166 (84)	3237 (85)
Black or African American	384 (10)	362 (9)
Asian	61 (2)	75 (2)
Native Hawaiian or Pacific Islander	10 (<1)	15 (<1)
Native American/Alaskan Native	18 (<1)	9 (<1)
Other	144 (4)	129 (3)
Received Prior Vaccination (≤ 2 years), n (%)	727 (19)	727 (19)

### Vaccine efficacy

The average attack rate for VMCCI over Seasons 1 and 2 was low at 23/3714 (0.6%) in the TIV group, and 45/3768 (1.2%) in the placebo group (Table 2). The VMCCI attack rate in the placebo group in Season 1 (1.7%) was lower than, but similar to, the assumption used for the original sample size estimate (2.0%). In Season 2, the attack rate for VMCCI in the placebo group was extremely low at 0.7% and was much lower than the assumed attack rate of VMCCI of 1.6% that had been used to determine the revised sample size for enrollment in Season 2.

**Table 2:** Vaccine efficacy against VMCCI, CCI, and LCI in the Per-Protocol analyses.

	Season 1 2005-2006		Season 2 2006-2007		Overall		Overall efficacy (97.5% CI lower bound)
		
	Vaccine N = 1706 n (%)	Placebo N = 1725 n (%)	Vaccine N = 2011 n (%)	Placebo N = 2043 n (%)	Vaccine N = 3714 n (%)	Placebo N = 3768 n (%)	
**VMCCI**	14 (0.8)	30 (1.7)	9 (0.4)	15 (0.7)	23 (0.6)	45 (1.2)	46.3% (9.8%)
**CCI**	19 (1.1)	38 (2.2)	11 (0.5)	22 (1.1)	30 (0.8)	60 (1.6)	49.4% (20.3%)
**LCI**	24 (1.4)	69 (4.0)	20 (1.0)	53 (2.6)	44 (1.2)	122 (3.2)	63.2% (48.2%)

VMCCI, culture-confirmed influenza A and B matching vaccine; CCI, any culture-confirmed influenza A and B; LCI, any culture- or serology-confirmed influenza A or B; CI, Confidence Interval

The average efficacy of TIV for the prevention of VMCCI was 46.3%, with a one-sided 97.5% CI lower bound of 9.8%, thus the null hypothesis of efficacy ≤ 35% could not be rejected. Attack rates for CCI and LCI over Seasons 1 and 2 were higher than the attack rates of VMCCI, but were still relatively low. In the TIV and placebo groups, attack rates for CCI were 0.8% and 1.6%, respectively, and the average efficacy of TIV against CCI was 49.3% with a 97.5% CI lower bound of 20.3%. The attack rates for LCI in the TIV and placebo groups were 1.2% and 3.2%, respectively, and the average efficacy of TIV against LCI was 63.2% with a 97.5% CI lower bound of 48.2% (Table 2). The results of the efficacy analyses conducted using the ITI set were essentially identical to those using the PP set; differing by only 0.1% for each of the VMCCI, CCI, and LCI endpoints (data not shown).

The antigenic characteristics of the influenza A and B viruses isolated from study participants showed good general concordance with CDC surveillance data in the same years [23,24]. In both Seasons 1 and 2, there was limited antigenic drift among A/H3N2 strains, and efficacy against disease associated with H3N2 isolates differed minimally for vaccine-matched strains (VMCCI) and all strains (CCI), or between seasons. No A/H1N1 viruses were isolated in Season 1, and in Season 2, overall efficacy against A/H1N1 was low at 23.9%, although this was based on a very small number of cases (6 in the TIV group, 8 in the placebo group). In Season 1, in which a Yamagata-lineage B virus was used in the vaccine and all B virus isolates were found to be of the Victoria lineage, there was thus by definition no VMCCI, and observed efficacy against CCI for Victoria lineage viruses was 0. In Season 2, in which B/Malaysia/2506/04 (a Victoria-lineage virus) was used in the vaccine, the point estimate for efficacy against VMCCI due to influenza B viruses was 100%, while the point estimate for efficacy against disease associated with Yamagata-lineage B viruses was 66% (estimates again based on small case numbers, with broad 95% confidence bounds).

The average efficacy of TIV against VMCCI was slightly higher in the 18-34 years stratum at 51.6% (97.5% CI lower bound 44.5%), than in the 35-49 years stratum at 44.5% (97.5% CI lower bound -11.8%), but this difference was not statistically significant (p = 0.79). Unexpectedly, the average efficacy against VMCCI was much lower in women, 19.4% (97.5% CI lower bound -42.8%), than in men, 89.0% (97.5% CI lower bound 53.0%; p value for the male:female contrast 0.0123). A similar, although slightly less marked trend between sexes was observed for the average efficacy of CCI and LCI (data not shown). Overall, women in this trial were marginally older than men (mean ± SD: women 33.3 ± 9.14 years; men 31.7 ± 9.12 years) and had a slightly higher rate than men of recent (within ≤ 2 years) previous influenza vaccine (women, 21%; men, 16%; p < 0.0001); these differences were similar in the TIV and placebo groups.

In the PP set, ILI symptoms were reported by a total of 362/3714 (10%) participants in the TIV group, and 427/3768 (11%) participants in the placebo group. An ad hoc analysis was conducted to assess efficacy when the definition of ILI was restricted to include fever (defined by self-observed oral temperature ≥ 38°C). With this modification, the average efficacy of TIV against VMCCI was 58.0%, with a 97.5% CI lower bound of -21.5% (due to fewer cases), and the average efficacy of TIV against CCI was 66.8%, with a 97.5% CI lower bound of 7.2%.

### Immunogenicity

The PP immunogenicity set included 1514 randomly selected participants, of which 1298 received TIV and 216 received placebo. TIV was highly immunogenic in Seasons 1 and 2 (Table 3). Immune responses were similar in both seasons for the A/H1N1 and the B virus components. HI GMTs increased 9.94-10.96-fold after immunization for the A/H1N1 component (A/NewCaledonia/20/99 in both seasons), with 68% seroconversion rates in each season. GMTs increased 11.45-12.36-fold for the B virus components, with 82% seroconversion to B/Jiangsu/10/03 (a Yamagata lineage virus) in Season 1 and 74% seroconversion to B/Malaysia/2506/04 (a Victoria lineage virus) in Season 2. Responses differed between seasons for the A/H3N2 component, with a stronger response in Season 1 to the A/New York/55/2004 antigen (15.84 fold-rise in GMT and 85% seroconversion rate) than in Season 2 to the A/Wisconsin/67/2005 antigen (10.59 fold-rise in GMT and 72% seroconversion rate). Despite this difference, the two-sided 95% CI lower bound for the proportions of active vaccine recipients attaining post-immunization HI titers ≥ 1:40 were ≥ 90% for all vaccine strains in both seasons.

**Table 3:** Summary of immunogenicity data in the immunogenicity set.

	Season 1 2005-2006		Season 2 2006-2007	
	
	Vaccine N = 891	Placebo N = 136	Vaccine N = 407	Placebo N = 80
**A/New Caledonia (H1N1)**				
Day 0 GMT (95% CI)^†^	35.2 (32.0, 38.6)	31.5 (24.7, 40.1)	35.5 (30.8, 40.9)	34.1 (24.8, 47.0)
Day 21 GMT (95% CI)^†^	385.4 (353.0, 420.8)	30.9 (24.7, 38.7)	352.5 (309.7, 401.3)	38.1 (28.4, 51.0)
GMFR (95% CI)‡	10.96 (9.87, 12.18)	0.98 (0.75, 1.28)	9.94 (8.55, 11.55)	1.11 (0.79, 1.56)
Seroconversion, n (%; 95% CI)	605 (68%; 65.0, 71.0)	0 (0%, 00.0, 00.0)	275 (68%; 63.0, 72.0)	1 (1%; 00.0, 4.0)
Day 0 HI titers ≥ 1:40, n (%; 95% CI)¶	454 (51%; 48.0, 54.0)	70 (51%; 43.0, 60.0)	200 (49%; 44.0, 54.0)	40 (50%; 39.0, 61.0)
Day 21 HI titers ≥ 1:40, n (%; 95% CI)¶	863 (97%; 96.0, 98.0)	71 (52%; 44.0, 61.0)	399 (98%; 97.0, 99.0)	41 (51%; 40.0, 62.0)

**A/New York (H3N2)**				
Day 0 GMT (95% CI)	16.3 (15.2, 17.5)	16.2 (13.4, 19.5)	-	-
Day 21 GMT (95% CI)†	258.4 (237.0, 281.7)	16.5 (13.2, 20.5)	-	-
GMFR (95% CI)‡	15.84 (14.56, 17.23)	1.02 (0.82, 1.26)	-	-
Seroconversion, n (%; 95% CI)¶	756 (85%; 82.0, 87.0)	1 (1) (1.%; 0.0, 2.0)		
Day 0 HI titers ≥ 1:40, n (%; 95% CI)	273 (31%; 28.0, 34.0)	41 (30%; 22.0, 38.0)	-	-
Day 21 HI titers ≥ 1:40, n (%; 95% CI)	837 (94%; 92.0, 96.0)	42 (31%; 23.0, 39.0)	-	-

**A/Wisconsin (H3N2)**				
Day 0 GMT (95% CI)	-	-	14.9 (13.3, 16.7)	14.2 (11.0, 18.4)
Day 21 GMT (95% CI)†	-	-	157.6 (140.3, 177.1)	14.7 (11.3, 19.1)
GMFR (95% CI)‡	-	-	10.59 (9.26, 12.11)	1.04 (0.77, 1.40)
Seroconversion, n (%; 95% CI)¶	-	-	292 (72%; 67.0, 76.0)	1 (1%; 0.0, 4.0)
Day 0 HI titers ≥ 1:40, n (95% CI)	-	-	116 (29%; 24.0, 33.0)	23 (29%; 19.0, 0.39)
Day 21 HI titers ≥ 1:40 (95% CI)	-	-	375 (92%; 90.0, 95.0)	23 (29%; 19.0, 39.0)

**B/Jiangsu (Yamagata)**				
Day 0 GMT (95% CI)†	25.4 (23.4, 27.4)	23.9 (19.6, 29.3)		
Day 21 GMT (95% CI)†	313.5 (290.1, 338.7)	24.5 (20.1, 29.9)		
GMFR (95% CI)‡	12.36 (11.35, 13.46)	1.02 (0.82, 1.27)	-	-
Seroconversion, n (%; 95% CI)¶	727 (82%; 79.0, 84.0)	1 (1%; 0.0, 2.0)		
Day 0 HI titers ≥ 1:40, n (%; 95% CI)	416 (47%; 43.0, 50.0)	63 (46%; 38.0, 55.0)		
Day 21 HI titers ≥ 1:40, n (%; 95% CI)	873 (98%; 97.0, 99.0)	65 (48%; 39.0, 56.0)		

**B/Malaysia (Victoria)**				
Day 0 GMT (95% CI)†			23.9 (19.6, 29.3)	35.7 (27.2, 46.8)
Day 21 GMT (95% CI)†			294.5 (263.9, 328.7)	38.4 (30.0, 49.2)
Seroconversion, n (%; 95% CI)¶	-	-	301 (74%; 70.0, 78.0)	1 (1%; 0.0, 4.0)
GMFR (95% CI)‡	-	-	11.45 (9.98, 13.13)	1.08 (0.79, 1.47)
Day 0 HI titers ≥ 1:40, n (%; 95% CI)¶	-	-	179 (44%; 39.0, 49.0)	45 (56%; 45.0, 97.0)
Day 21 HI titers ≥ 1:40, n (%; 95% CI)¶	-	-	396 (97%; 96.0, 99.0)	48 (60%; 49.0, 71.0)

GMT, geometric mean titer; GMFR geometric mean fold ratio; HI, hemagglutination-inhibiting; CI, Confidence Interval

^† ^95% CI based on log (base 10) transformed reciprocal titers, error term from ANOVA

^‡ ^Two-sided, 95% CI on the geometric mean fold-increase in reciprocal HAI antibody titer based on the log (base 10) transformed data

^¶ ^Two-sided, 95% CI on the point estimates for binomial proportions

Treatment with placebo had a negligible effect on HI titers in both seasons. In view of the unexplained gender difference in efficacy noted above, a post-hoc examination of immune response by gender was performed. The seroconversion rate for the A/New Caledonia H1N1 antigen was lower among women than men in both seasons, and lower GMTs for this virus were found among women (318.6) compared with men (414.6) in Season 2; however, the proportion of men and women with A/New Caledonia/20/99 HI titers ≥ 1:40 was ≥ 96% in both seasons, and there were no other immunogenicity differences of note between men and women (Table 4).

**Table 4:** Summary of immunogenicity in men and women receiving TIV in the immunogenicity set.

Year	Antigen	Parameter	Men value (95% CI) N = 482	Women value (95% CI) N = 816
Season 1	A/H1N1	% Seroconversion	72 (67, 77)	66 (62, 70)
2005-6		Day 21 GMT	385.9 (334.6, 445.1)	385.1 (344.4, 430.7)
		Day 21% ≥ 40	98 (96, 99)	96 (95, 98)
	A/H3N2	% Seroconversion	85 (81, 89)	85 (82, 88)
		Day 21 GMT	265.4 (231.0, 305.0)	254.4 (227.7, 284.2)
		Day 21% ≥ 40	95 (93, 97)	93 (91, 95)
	B	% Seroconversion	83 (79, 87)	81 (77, 84)
		Day 21 GMT	329.6 (291.3, 373.0)	304.6 (275.8, 336.4)
		Day 21% ≥ 40	99 (98, 100)	98 (96, 99)

Season 2	A/H1N1	% Seroconversion	72 (65, 79)	65 (59, 71)
2006-7		Day 21 GMT	414.2 (337.0, 508.9)	318.6 (269.7, 376.3)
		Day 21% ≥ 40	99 (97, 100)	98 (96, 99)
	A/H3N2	% Seroconversion	67 (60, 74)	75 (69, 80)
		Day 21 GMT	141.9 (117.7, 171.1)	168.3 (145.1, 195.3)
		Day 21% ≥ 40	90 (86, 95)	93 (90, 96)
	B	% Seroconversion	73 (66, 80)	74 (69, 80)
		Day 21 GMT	290.9 (244.8, 345.7)	296.8 (257.2, 342.5)
		Day 21% ≥ 40	97 (95, 100)	97 (95, 99)

GMT, geometric mean titer

In Season 1, immunogenic lot consistency based on GMT values was demonstrated using the Z_min _statistic by the method of Wiens and Iglewciz [22], and according to the study protocol, was not retested in Season 2 (data not shown).

### Reactogenicity and safety

One or more solicited events after immunization occurred in 2487/3783 (66%) participants in the TIV group and 1675/3828 (44%) participants in the placebo group (p < 0.0001) (Table 5). Common reactogenicity complaints (30 minutes post-vaccination through Day 3) that were significantly more frequent in the TIV versus the placebo group included pain/soreness at the injection site (51% and 14%, respectively), tiredness (20% and 18%, respectively), and myalgia and/or arthralgia (18% and 10%, respectively). Less frequent, but also significantly associated with active treatment, were malaise and redness and/or swelling at the injection site. Oral temperatures of ≥ 37.5°C/99.9°F were reported by 3% of TIV recipients and by 1% of placebo recipients (p = 0.0005); however, the great majority of these reports included maximal temperatures of ≤ 38.5°C/101.3°F. From Day 0 through Day 3 post-vaccination, there was no significant association between TIV vaccination and reports of headache, red eyes, cough, sore throat, and hoarseness or pain on swallowing.

**Table 5:** Incidence of TIV reactogenicity events from Day 0^† ^to Day 3 in the Safety Set

Symptom	TIV N = 3783 n (%)	Placebo N = 3828 n (%)	P-value^¶^
At least 1 vaccine reactogenicity event	2487 (66)	1675 (44)	<0.0001
Fever^‡^	96 (3)	55 (1)	0.0005
Injection site pain/soreness	1933 (51)	530 (14)	<0.0001
Injection site redness	475 (13)	234 (6)	<0.0001
Injection site swelling	418 (11)	109 (3)	<0.0001
Myalgia and/or arthralgia	692 (18)	389 (10)	<0.0001
Headache	683 (18)	716 (19)	0.5491
Tiredness	761 (20)	678 (18)	0.0049
Chills	158 (4)	136 (4)	0.1526
Malaise	338 (9)	236 (6)	<0.0001
Red eyes	250 (7)	231 (6)	0.2772
Swelling of the face	51 (1)	37 (<1)	0.1327
Cough	286 (8)	250 (7)	0.0719
Chest tightness or difficulty in breathing	128 (3)	107 (3)	0.1274
Sore throat, hoarseness or pain on swallowing	324 (9)	344 (9)	0.5689

^†^>30 minutes post-vaccination

^¶^Fisher's Exact test (p < 0.05 considered significant)

^‡^Temperature ≥37.5°C/99.5°F

In both groups, the most frequent unsolicited AEs from Days 0 through Day 21 with TIV and placebo were pharyngolaryngeal pain (120/3783 and 120/3828, respectively [both 3%]), headache (118/3783 and 121/3828, respectively [both 3%]), and fatigue (108/3783 and 120/3828 [both 3%]). Overall, the rate of AE reports in the TIV group was 808/3783 (21%) and in the placebo group was 736/3828 (19%) (p = 0.021).

From Day 0 to Final Visit, the incidence of unsolicited AEs by SOC reported by ≥ 1% of participants was higher with TIV than placebo, with injection site pain (95/3785 [3%] and 30/3828 [1%], respectively; p < 0.0001), and injection site erythema (45/3783 [1%] and 9/3828 [<1%], respectively; p < 0.0001) accounting for the between-group difference. No other significant differences were seen between groups for incidence rates of AEs by SOC.

Unsolicited SAEs were uncommon, and were reported by 1% of participants in both treatment groups. Overall, 44/3783 (1%) and 39/3728 (1%) of participants in the TIV and placebo groups, respectively, experienced at least 1 SAE. All SAE types occurred in ≤ 1% of participants. None of the SAEs were considered by the investigators to be vaccine-related. One subject in the placebo group had a fatal road traffic accident during the study. Severity of ILI was not systematically assessed; a single subject in the placebo group was hospitalized with a diagnosis of influenza.

### Pregnancies

There were a total of 25 pregnancies in the TIV group, and 32 in the placebo group, of which 4 (16%: 95% CI; 5%, 36%) and 7 (22%: 95% CI; 9%, 40%), respectively, ended in spontaneous abortion. Three pregnancies were electively terminated. One placebo recipient was induced at 34 weeks because of intrauterine growth retardation, and the infant was reported to have 2 hernias, which were surgically repaired, and a heart murmur. Two pregnant participants were lost to follow up, and there were 18 (72%) and 22 (69%) full-term births with healthy infants in the TIV and placebo groups, respectively.

## Discussion

Although inactivated influenza vaccines are recommended for many populations, estimates as to their true efficacy vary widely. It is commonly held that the efficacy of TIVs is 60 to 80% in healthy younger adults and similar in children over the age of 2 years, but less in the elderly (particularly the chronically-ill or institutionalized) [25-29]. The degree of antigenic match between the vaccine and circulating virus strains, the exact illness endpoint used, and the surveillance methodology in clinical trials can influence efficacy estimates. Efficacy data concerning children under 2 years of age are very limited.

This placebo-controlled, randomized study was conducted to assess the efficacy, safety, and immunogenicity of a TIV over the 2005 2006 (Season 1) and 2006 2007 (Season 2) influenza seasons in healthy adults in the US. The primary endpoint was protection against VMCCI, as requested by US regulatory authorities, rather than the frequently cited endpoints of CCI or LCI. Although the LCI endpoint has been questioned by some authors [15], it is still widely used, and constitutes the primary endpoint for the majority of the published TIV efficacy experience.

Despite a good degree of 'vaccine-match' among the influenza isolates in the trial (75.5%), and a strong immune response to the vaccine in both years, the average efficacy over Seasons 1 and 2 of TIV against VMCCI was only 46.3%, with a one-sided 97.5% CI lower bound of 9.8%. While the vaccine was clearly efficacious relative to placebo (one-sided 97.5% CI lower bound for efficacy excluded 0), these results did not satisfy the pre-defined criterion for success, i.e. exclusion of efficacy of ≤ 35% (as evidenced by a lower 97.5% CI of >35%). The results were similar for CCI, with an average efficacy of 49.4%, with one-sided 97.5% CI lower bound of 20.3%, the CI becoming narrower due to the greater number of cases.

Efficacy was higher for the LCI endpoint, at 63.2%, with one-sided 97.5% CI lower bound of 48.2%; this is generally consistent with previously reported TIV efficacy estimates in studies using this endpoint [12,14,30]. While an intrinsic bias of the LCI endpoint in favor of the vaccine has been noted [15,16] and, therefore, limits its utility for regulatory purposes, it should also be noted that this endpoint may improve sensitivity relative to culture. In addition, the endpoint of LCI has been used in many prior clinical trials of TIV, yielding estimates of TIV efficacy in adults of about 70% [14,15]. Thus, the estimated vaccine efficacy against LCI in this trial is consistent with that estimated in prior trials of other TIVs.

A key factor influencing efficacy findings in influenza vaccine studies is the disease attack rate, and the unpredictable nature of epidemic intensity poses a challenge when planning influenza vaccine efficacy trials [12,13,25,31]. Our study was powered on the assumptions that vaccine efficacy would approximate 70%, based on the prior literature reporting culture-confirmed influenza endpoints, and that the attack rate for CCI in the placebo group would be 2.0% across both seasons. The attack rate assumption was adjusted in Season 2 to 1.6% based on the regulatory request to adopt VMCCI as the primary endpoint, and the sample size correspondingly increased. The actual VMCCI attack rate in placebo recipients in Season 1 was close to our prediction, but in Season 2 the attack rate was less than half of that predicted, and this had a large negative impact on the precision of the average efficacy estimate. Despite this, it was also clear that the efficacy observed did not differ markedly between the seasons, and might not have sufficed to exclude the 35% lower bound of the primary hypothesis even if the VMCCI attack rates in Season 2 had matched those in Season 1.

Similar to the findings of our study, the Centers for Disease Control and Prevention surveillance data summaries for the 2005-2006 and 2006-2007 influenza seasons in the US suggest that these were relatively mild influenza seasons [23,24]. The weekly percentage of outpatient visits to US sentinel providers for ILI peaked twice in the 2005-2006 season at 3.3% and 3.2% (baseline 2.2%), and peaked twice in the 2006-2007 season at 3.0% and 3.5% (baseline 2.1%) [23,24]. In the prior three seasons, the peak percentage of outpatient visits for ILI had ranged from 2.3% to 7.6% [23,24].

A negative impact of low attack rates in a given season on point estimates of efficacy has been well documented. Previous studies of a TIV product, involving the same investigators, methods, and source populations over successive seasons, have reported dramatic differences in efficacy estimates between seasons, without obvious explanation based on circulating strain match to the vaccine [12,13]. In these studies, the low efficacy estimates were found in seasons with lower overall attack rates [12,13]; a similar experience has occurred in pediatric trials [31]. While a clear reason for this phenomenon is not apparent, it can be speculated that low background attack rates may result from either relatively high pre-existing population resistance to the circulating strain(s), or modest intrinsic virulence of the circulating strains, such that only intense exposures lead to disease. In either case, the attainable impact of a vaccine might be blunted. Primary detection of influenza virus infections in this study was by means of viral culture; with the use of molecular methods confined to characterization of isolates. Other reports have suggested that the use of RT-PCR as a primary detection tool may improve the rate of ascertainment of influenza virus infections by 20 to 30% [12,13,31,32], but this maneuver does not appear, in and of itself, to have a major impact on point estimates of efficacy.

As suggested previously, a further factor that may compromise vaccine efficacy is the extent of antigenic drift and the degree of match between vaccine and circulating viruses [11,12,33]. In our study, the majority of cases in both seasons were due to influenza A/H3N2 viruses, and antigenic drift was modest and had little apparent impact. However, only 5/17 cases of culture-confirmed influenza B disease were due to viruses of the same lineage as the vaccine strain in the relevant season (data not shown), and average efficacy against non-matching B strains was low (~16%); this result clearly reduced the estimate of efficacy against all CCI.

The definition of influenza-like illness (ILI) in our study required that illness impeded normal daily activities, with cough plus ≥ 1 other symptom from a panel including both respiratory and systemic complaints, so this allowed for a diagnosis without need for a systemic symptom (i.e. fever, myalgia/arthralgia). Cough has been previously found to be one of the most sensitive clinical indicators of influenza, but has little specificity, whereas fever is a classically-cited characteristic of influenza, with substantial positive predictive value, but lower sensitivity [34]. Fever was not made a requirement for ILI in our study in order to maximize sensitivity. However, our ad hoc analyses showed that narrowing of the case definition to require fever reduced the case rate but notably improved the apparent efficacy of TIV. A potential interpretation of this finding may be that TIV is most effective against severe manifestations of influenza, and less so against illnesses with predominantly respiratory symptoms. Although efficacy against severe disease is desirable, the relatively sensitive but non-specific case definition in our study may have allowed for the inclusion of respiratory illness caused by mixed infections in which influenza viruses may have been minor or coincidental. An additional factor that could have compromised the results is the highly decentralized source of the specimens, and the transportation of specimens to a centralized laboratory may not have been optimal for the recovery of influenza viruses from nasopharyngeal swabs.

An unexpected finding of the study was the difference between men and women for the primary endpoint: men, 89.0%; and women, 19.4%. Women were slightly older than men (33.3 and 31.7 years, respectively), and were more likely to have had previous recent influenza vaccination (21% and 16%, respectively). These between-gender differences were reflected in both treatment groups, which were well matched for all baseline characteristics. In addition, all three viruses were culture-confirmed in men and women, there was no differential temporal clustering of cases, and serum HI titers did not differ meaningfully between the sexes (data not shown); this is in contrast to a previous large study of a TIV, which reported significantly higher GMT responses in women than men, regardless of age or dose or influenza strain [35]. The attack rate of VMCCI among placebo-treated men and women was similar (1.27% and 1.14%, respectively), which suggests that both sexes had a similar level of exposure. At present, the difference in values between men and women remains unexplained and must be viewed with caution because a further subdivision of the already-small case numbers results in very broad CIs about the efficacy estimates.

In terms of seroconversion and rates of attainment of post-vaccinal HI reciprocal titers ≥ 40 ("seroprotection" rates), the TIV in this study fulfilled the immunogenicity criteria for the accelerated approval of seasonal influenza vaccines established by the US FDA Center for Biologics Evaluation and Research for all strains in both seasons [36]. In addition, manufacturing consistency was confirmed for three consecutive TIV lots in the 2005-2006 season, based on GMT values. Vaccine immunogenicity as measured by induction of hemagglutination-inhibiting antibodies provides a marker of potential vaccine effectiveness in the absence of viral circulation and has therefore been, and continues to be, a useful tool for the registration of influenza vaccines. Nevertheless, definitive demonstration of a single antibody titer which constitutes an absolute correlate of protection for all virus strains remains elusive, and the current study was not designed to demonstrate this.

The reactogenicity events reported in the study were consistent with those commonly reported with TIVs. Reactogenicity events that were significant in the TIV versus placebo group included injection site pain, injection site redness and swelling, myalgias, arthralgias, fever and fatigue; the majority of events were mild (grade 1) in severity. There was a slightly higher incidence of spontaneous AEs in the TIV versus placebo group reported up to 21 days post-vaccination, and this was primarily due to the persistence of injection site pain and redness. Overall, the results suggest that the safety profile of TIV was acceptable, and consistent with the historical performance of similar products.

## Conclusions

This randomized, placebo-controlled trial showed that the average efficacy of TIV for the prevention of VMCCI over the 2005-2006 and 2006-2007 influenza seasons was 46.3%. Although the differences between the vaccine and placebo groups in the primary endpoint did not meet the pre-defined criterion for success, the TIV clearly demonstrated clinical benefit (i.e. it was readily differentiated from placebo by all culture and/or laboratory-confirmed influenza endpoints) and the primary endpoint result must be interpreted with some caution in view of the very low influenza attack rates in both seasons; a factor which has previously been associated with low efficacy estimates in influenza vaccine trials. Furthermore, the immune responses to TIV fulfilled the licensure criteria for seasonal influenza vaccines [36]. Overall, TIV had a safety profile that was considered to be acceptable, and was consistent with other inactivated influenza vaccines.

## Competing interests

GSK Biologicals was the funding source and was involved in all stages of the study conduct and analysis. GSK Biologicals also took in charge all costs associated with the development and the publishing of this manuscript. The corresponding author had full access to the data, and final responsibility for submission of the manuscript for publication.

LJ has received research funding from manufacturers of influenza vaccines, including GSK, Sanofi Pasteur, and Novartis and has served as a consultant to GSK and Novartis. Dr Harry Keyserling received grant support from GlaxoSmithKline to conduct the study. JB has no conflict of interest to declare. NB is a former employee of GlaxoSmithKline Biologicals and reports ownership of equity or stock options. LFF is a full-time employee of GlaxoSmithKline. JT discloses having received laboratory support from GSK and MerciaPharma, as well as clinical trial support from Protein Sciences Corporation, Vaxinnate, Ligocyte, Wyeth, Bavarian Nordic, Sanofi and PaxVax. JT discloses scientific advisory board activities for Immune Targeting Systems and Toyama Chemical Concern.

## Authors' contributions

All authors participated in the design, implementation, analysis and interpretation of the study. All authors read and approved the final manuscript. LF and NB were involved in all phases of the study, and led the clinical team at GSK Biologicals. LAJ, MJG and HLK led the clinical team at their respective centers, JB conducted the data analysis. JJT managed the team responsible for detailed antigenic characterization and vaccine match analysis.

## Pre-publication history

The pre-publication history for this paper can be accessed here:

http://www.biomedcentral.com/1471-2334/10/71/prepub
